# The Vineyard Project: exploring new paths toward community integration and competitive employment

**DOI:** 10.1192/j.eurpsy.2023.692

**Published:** 2023-07-19

**Authors:** A. Barbieri, E. Rossero

**Affiliations:** ^1^Mental Health Department, ASL CN1, Cuneo; ^2^Eclectica+ Research and Training, Turin, Italy

## Abstract

**Introduction:**

Employment as a critical domain of functioning is an important target of recovery-oriented programs for people with psychiatric disabilities. Evidence shows that persons participating in competitive employment which meets their vocational needs are more likely than people in sheltered work programs to feel included in their communities, to report satisfaction with work and a high quality of life.

**Objectives:**

The Vineyard Project is a program engaging young people with different forms of mental ill-health in local practices of hand-harvesting grape. The program stems from a pilot carried out in September 2022 with a group of people aged 16-25, who worked with professional vine growers in the renowned area of Langhe (Italy). Aims were manifold: i) for the group: involvement in a culturally meaningful activity that is part of the transformative process of winemaking, as a way to overcome social anxiety symptoms and poor self-efficacy; ii) for the community: to attempt overcoming structural stigma that may undermine an employer’s willingness to hire a person with a psychiatric diagnosis; iii) to develop an evidence-based rehabilitation program aimed at competitive employment.

**Methods:**

To foster community integration, the program was hosted in a real-world setting. The program is multidisciplinary, involving psychiatrists, psychologists, rehabilitation specialists and sociologists. Clinical assessment and semi-structured interviews with participants are performed.

**Results:**

Preliminary research findings provide evidence to develop the program according to the following principles: on-site practical training provided in a 1:1 relationship with professional workers (i.e. natural supports), supplemented by extra educational resources available in the community (e.g. local School of Enology); covering of all the vineyard activities throughout the year (pruning, binding, harvesting…); growth of expertise on an individual as well as on a group level, to foster the building of a cohesive team that can compete on the labour market and that provides participants with a sense of membership and identity; opportunity for new participants to join the team on an annual basis, acknowledging their peers as experts who can in turn pass on their knowledge; continual assessment of the ever-changing needs of participants and qualitative inquiry of their perspective, to provide time-unlimited support and ongoing adjustment of the program.

**Conclusions:**

The Vineyard Project aims to eventually establish a rehabilitation tool, resulting from the combination of multi-disciplinary approaches, that can be tested and applied to work settings different from the viticultural environment where it had its origin.

**Disclosure of Interest:**

None Declared

